# Synthesis of 2D layered transition metal (Ni, Co) hydroxides via edge-on condensation

**DOI:** 10.1038/s41598-024-53969-2

**Published:** 2024-02-15

**Authors:** Lu Ping, Gillian E. Minarik, Hongze Gao, Jun Cao, Tianshu Li, Hikari Kitadai, Xi Ling

**Affiliations:** 1 Division of Materials Science and Engineering, Boston University, 15 St. Mary’s Street, Boston, MA 02215 USA; 2 Department of Chemistry, Boston University, 590 Commonwealth Avenue, Boston, MA 02215 USA; 3 The Photonics Center, Boston University, 8 St. Mary’s Street, Boston, MA 02215 USA

**Keywords:** Chemistry, Engineering, Materials science, Physics

## Abstract

Layered transition metal hydroxides (LTMHs) with transition metal centers sandwiched between layers of coordinating hydroxide anions have attracted considerable interest for their potential in developing clean energy sources and storage technologies. However, two-dimensional (2D) LTMHs remain largely understudied in terms of physical properties and applications in electronic devices. Here, for the first time we report > 20 μm α-Ni(OH)_2_ 2D crystals, synthesized from hydrothermal reaction. And an edge-on condensation mechanism assisted with the crystal field geometry is proposed to understand the 2D intra-planar growth of the crystals, which is also testified through series of systematic comparative studies. We also report the successful synthesis of 2D Co(OH)_2_ crystals (> 40 μm) with more irregular shape due to the slightly distorted octahedral geometry of the crystal field. Moreover, the detailed structural characterization of synthesized α-Ni(OH)_2_ are performed. The optical band gap energy is extrapolated as 2.54 eV from optical absorption measurements and the electronic bandgap is measured as 2.52 eV from reflected electrons energy loss spectroscopy (REELS). We further demonstrate its potential as a wide bandgap (WBG) semiconductor for high voltage operation in 2D electronics with a high breakdown strength, 4.77 MV/cm with 4.9 nm thickness. The successful realization of the 2D LTMHs opens the door for future exploration of more fundamental physical properties and device applications.

## Introduction

The first successful exfoliation of graphene in 2004 sparked a dramatic increase in two-dimensional (2D) materials research and the repertoire of reported 2D materials family has since expanded. 2D crystals composed of single- or few-layers of atoms display extraordinary chemical, optical, and electronic properties compared with their bulk 3D counterparts due to quantum confinement effect^1^. Nevertheless, only several 2D materials such as graphene^2^, transition metal dichalcogenides^3^, and hexagonal boron nitride^4,5^, are reasonably well-studied for 2D electronic and optoelectronic applications, thanks to the development of successful synthesis methods for large domains and films. Among the over thousands of van der Waals 2D materials^6,7^, unfortunately, many of them are underexplored experimentally, due to the lack of proper synthesis methods for sufficiently large domains. For example, layered transition metal hydroxides (LTMHs) are a group of materials with van der Waals layered structures, but not well studied as 2D electronic materials so far. Several synthetic methods have been proposed to achieve 2D LTMHs crystals including chemical precipitation^8,9^, electrochemical precipitation^10,11^, chemical aging^12^, sol–gel^13^, hydrothermal^14^, solvothermal methods^15^, and top-down exfoliations^16^. The obtained materials have demonstrated great capability as high-performance electrocatalysts for oxygen evolution reaction (OER)^17,18^ and high-capacity electrode materials for supercapacitors^19,20^ thereby exhibiting great potential for applications in terms of clean energy^21–23^ and energy storage^24–26^. However, the reported 2D LTMHs are usually nanosheets^13,16^, nanoplates^27,28^, nanoflowers^29^ with domain size from hundreds of nm to a few µm. Larger lateral domains of 2D LTMHs (> 10 µm) that are desired for certain applications such as semiconductor devices have never been reported^30,31^. Thus, studies of their electronic and magnetic properties have been limited to theoretical investigation^32–34^.

In this work, we achieve largest ever reported 2D α-Ni(OH)_2_ single crystals (> 20 µm) and Co(OH)_2_ (> 40 µm) in a hydrothermal synthesis process. The 2D growth mechanism of LTMHs is established and its different exertion on different transition metals (Ni, Co) is discussed and explained by spin-state geometry theory. Specifically, [Ni(H_2_O)_6_]^2+^ that has perfect O_h_ symmetry, allows the new units adding on via the edge and limits the expansion of the crystals in two directions only. Additionally, a comprehensive experimental study is performed using the hydrothermal method to testify the 2D growth mechanism of LTMHs. Further characterizations on the structure and electric properties of the as synthesized 2D Ni(OH)_2_ reveal high crystallinity, mostly α phase, and bandgap of 2.5 eV with a high breakdown strength of 4.77 MV/cm on a 4.9 nm thick flake, showing a great potential as WBG semiconductor for high voltage operation. We anticipate this achievement will pave the way for the study of fundamental properties and applications of 2D LTMHs crystals in 2D micro-nanoelectronics devices, which is not accessible on reported LTMH samples with limited domain sizes.

## Results and discussion

### Synthesis and morphology characterization of 2D Ni(OH)_2_ and Co(OH)_2_

**Figure 1:**
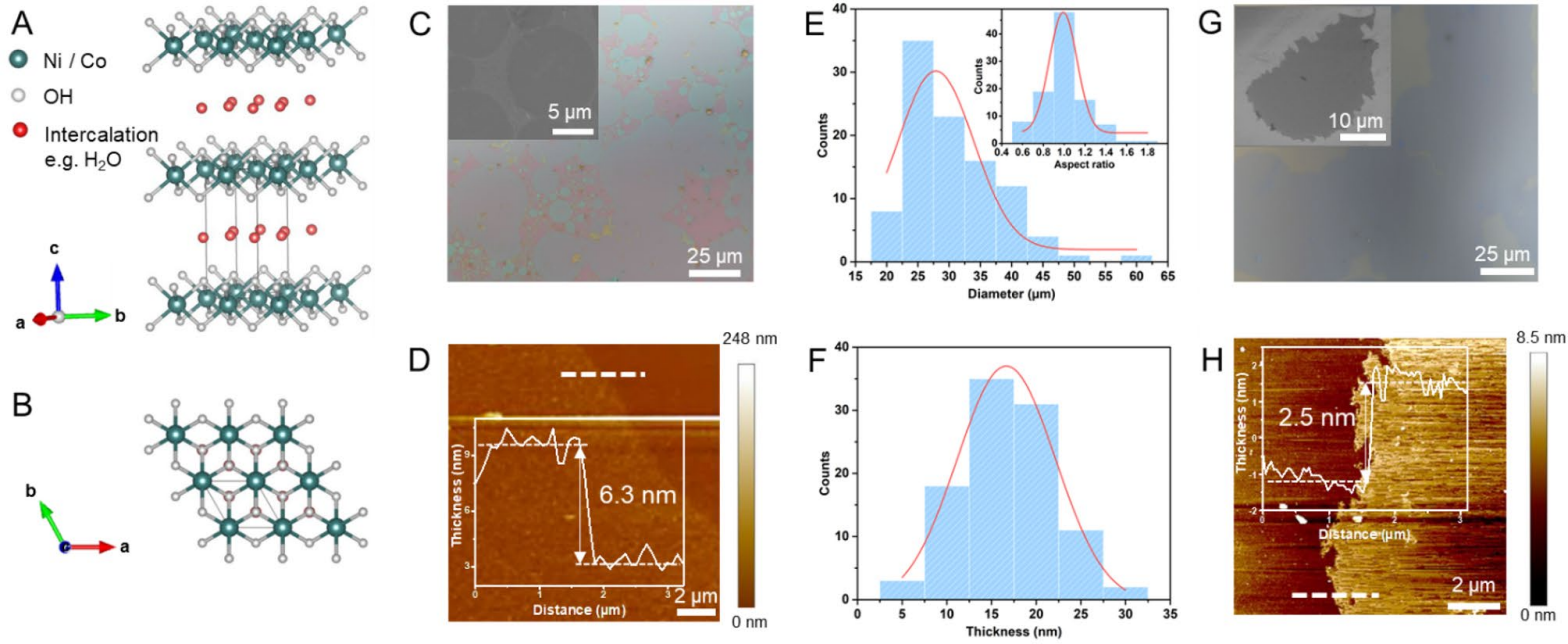
Crystal structure and morphology characterization of M-(OH)_2_, (M = Ni, Co) (**A**,**B**) Crystal structure of α-M(OH)_2_. (**A**) Side view; (**B**) Top view. (**C**) Optical image and SEM image (inset) of synthesized Ni(OH)_2_ flakes on SiO_2_/Si substrate. (**D**) AFM image of a Ni(OH)_2_ flake, the white dash line indicates the location where the thickness is measured. Inset: height profile along the white dash line. (**E**) Size distribution of 100 Ni(OH)_2_ flakes in a randomly chosen area. Inset: aspect ratio distribution, which is defined as the ratio of diameters measured along two perpendicular directions, indicating the shape of flakes. (**F**) Thickness distribution of 100 Ni(OH)_2_ flakes in a randomly chosen area. (**G**) Optical image of synthesized 2D Co(OH)_2_ flakes on SiO_2_/Si substrate, domain size reaches ~ 40 µm on one direction. Inset: SEM image of synthesized 2D Co(OH)_2_ on SiO_2_/Si substrate, showing the crystal domain is continuous. (**H**) AFM image of a Co(OH)_2_ flake, the white dash line indicates the location where the thickness is measured. Inset: height profile along the white dash line, showing the thickness of the flakes is 2.5 nm.

Figure 1A,B show the crystal structure of α-M(OH)_2_ (M = Ni and Co), which is a typical layered material with a “sandwich” structure of bivalent metals between two layers of octahedrally-coordinated OH^−^ anions. The intercalation of H_2_O between the layers expands the interlayer distance and distinguishes α phase from β phase, which has a smaller interlayer gap due to the absence of intercalation (Fig. S1). In a typical synthesis condition (more details in “Methods”), we perform the reaction at 120 °C with a cooling rate of 1.5 °C/min, which gives the most and largest 2D domains of Ni(OH)_2_ (Fig. 1C). The surface morphology is further characterized using scanning electron microscopy (SEM) (Fig. 1C inset), showing the flakes are continuous and smooth, despite small dis-uniformity probably due to thickness change or contaminations. Moreover, Fig. 1D shows the atomic force microscopy (AFM) image of a flake of 6.3 nm, corresponding to ~ 8 atomic layers^35^. More AFM images of large flakes are shown in Fig. S2, showing the continuous crystal domain in a large area. The average domain size and thickness is 20 μm (Fig. 1E) and 16 nm (Fig. 1F), respectively, through surveying 100 flakes in randomly chosen areas. Notably, this is the largest domain size of 2D Ni(OH)_2_ ever reported, which is also extraordinary compared to other 2D nanosheets typically synthesized using hydrothermal methods^36–40^. We also obtain the aspect ratio (dimensionless) by measuring the diameters of each flake along the two perpendicular directions. The plotted distribution (Fig. 1E inset) indicates about 50% of flakes have an aspect ratio of 1.0 and 84% of surveyed flakes maintain quasi-circular dimensions with aspect ratios within the range of 0.8–1.2. The (quasi-) circular shape observed visually is the consequence of the isotropic and outward radial crystal growth. Specifically, Ni^2+^ with its d^8^ configuration (Fig. S3) has an even number of electrons on degenerate orbitals, experiencing no distortion from Jahn–Teller (JT) theorem, which describes spontaneous symmetry breaking to provide energetic stabilization of transition metal complexes when there is an odd number of electrons occupying degenerate orbitals^41–43^. The perfect O_h_ symmetry on [Ni(H_2_O)_6_]^2+^ with identical bond length and electron density on all six Ni-H_2_O metal–ligand coordination bonds allows the formation of 2D crystals with large domain.

Following the same synthesis protocol, we also successfully obtain Co(OH)_2_ thin flakes with even larger domains (> 40 μm), but the shapes are random rather than circular and the density of such large domains is significantly lower than that of Ni(OH)_2_ (Fig. 1G). This is also the largest Co(OH)_2_ ultrathin domains (Fig. 1H) ever reported^44–46^ and the identification from X-ray photoelectron spectroscopy (XPS) is shown in Fig. S4. The surface morphology revealed by SEM (Fig. 1G inset) suggests the continuity of these crystals and shows the edge of Co(OH)_2_ is less tide compared with Ni(OH)_2_, which is caused by the high spin d^7^ configuration of [Co(H_2_O)_6_]^2+^. For [Co(H_2_O)_6_]^2+^ (Fig. S5), it suffers slight JT distortion and has a weak compression along the *z*-axis due to one vacancy in t_2g_ set, leading to D_4h_ symmetry^47^. The small degree of JT distortions leads to less perfect structural alignment when [Co(H_2_O)_6_]^2+^ units add onto the exiting seed crystals, which would result in distorted-quasi circular morphology, but the large domain size could still possibly be achieved (Fig. 1G). Another case of JT distortion is presented with [Cu(H_2_O)_6_]^2+^ (Fig. S6), where the high spin d^9^ configuration of Cu^2+^ leads to three electrons in the e_g_ set, and thus largely elongates along the *z*-axis^47,48^. While the elongated axial bonds in [Cu(H_2_O)_6_]^2+^ also give the complex D_4h_ symmetry, the degree of distortion is more pronounced owing to the fact that the e_g_ set are anti-bonding orbitals (Fig. S6A), which would likely to direct the growth on a singular direction (Fig. S6B), forming nanorods^49^ or nanobelt^50^. 

### Investigation of the 2D growth mechanism of Ni(OH)_2_

**Figure 2:**
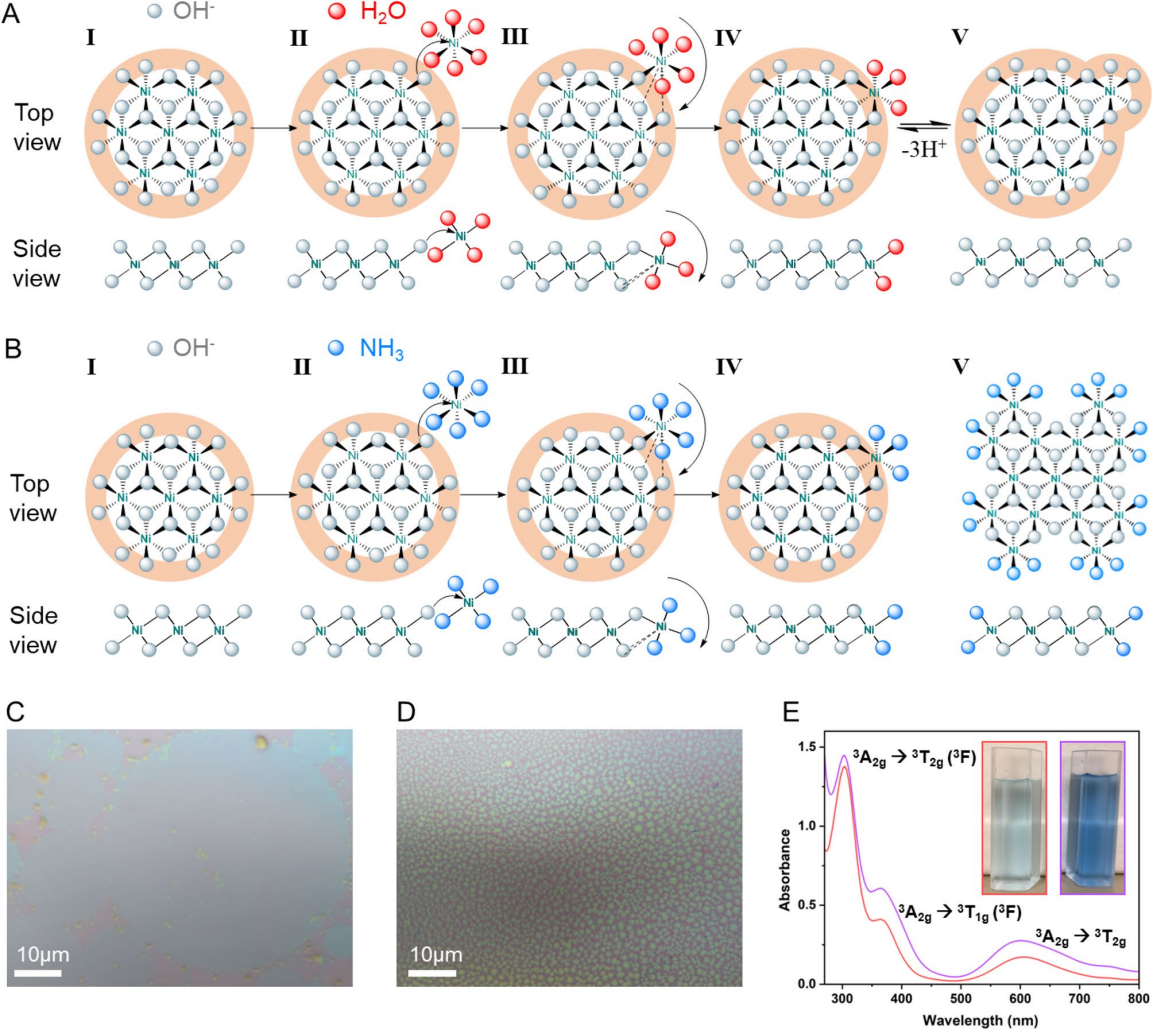
2D Ni(OH)_2_ growth mechanism. (**A**) 2D growth mechanism of Ni(OH)_2_ via edge-on condensation after nucleation. I. The buildup of localized negative charge (orange ring) incurred by edge OH^−^ ions on the nucleus attracts approach of Ni^2+^ ions; II. Ligand exchange at labile Ni^2+^ centers, undergoing substitution with hanging OH^−^ groups when proximal to an existing crystal, which guides continues substitution of terminal OH^−^ groups at newly bounded Ni^2+^; III. The unit is locking into the crystal structure; IV. Deprotonation of coordinated H_2_O or its displacement by OH^−^ to extend the crystal domain (V) or to continue the add-on (II). (**B**) Schematic of [Ni(NH_3_)_6_]^2+^ blocking the growing nuclei and crystals, the orange ring represents the localized negative charge. (**C**) Optical image of the obtained 2D Ni(OH)_2_ without additional NH_4_OH, where circular flakes are widely observed. (**D**) Optical image of the obtained 2D Ni(OH)_2_ with 0.6 ml additional NH_4_OH, the crystal size are significantly decreased. (**E**) Absorbance of 2D Ni(OH)_2_/H_2_O dispersion without (red curve) and with 0.6 ml additional NH_4_OH (purple curve), three absorption bands assigned left to right as ^3^A_2g_ → ^3^T_1g_ (^3^F), ^3^A_2g_ → ^3^T_1g_ (^3^F), ^3^A_2g_ → ^3^T_2g_, consistent with homoleptic six-coordinate Ni(II) complexes. Inset: 2D Ni(OH)_2_/H_2_O dispersion of (**C**) (left) and (**D**) (right).

During the synthesis, seed crystals formed by a few Ni^2+^ ions with edge hydroxyl ions and localized negative charge (Fig. 2A I) initiate the 2D growth^51^. In order for a single crystal to maintain electrical neutrality, the negative charged seed crystal attracts more Ni^2+^ ions to approach (Fig. 2A II). Meanwhile, the “hanging” water of solvated [Ni(H_2_O)_6_]^2+^ units formed in aqueous solution will be substituted by hydroxyl groups rapidly and chemical bonds will be built between seed crystal and [Ni(H_2_O)_6_]^2+^ units in the solution since the [Ni(H_2_O)_6_]^2+^ is a stable yet labile species. Once this association has been established, continued substitution will occur at the newly bound metal ion (Fig. 2A III) until it is pulled down into the crystal structure (Fig. 2A IV). The terminal H_2_O ligands further deprotonate to form new hanging hydroxy groups and bind with new approaching [Ni(H_2_O)_6_]^2+^ units (Fig. 2A V). Since each surface OH^−^ of seed crystal is already bonded with three Ni^2+^ and have fully incorporated into the crystal structure (Fig. 2A), the free moving [Ni(H_2_O)_6_]^2+^ units tend to approach these intra-planar “hanging” ligands at the edge that have strong localized negative charge. Thus, the expansion is limited to two dimensions only, leading to the formation of 2D structure.

In order to testify the mechanism, we investigated the influence of several parameters to the synthesis including the temperature, cooling rate and pH. The detailed discussion can be found in Supplementary Information (Sect. 1). Here we focus on the discussion of the role of NH_3_ to support the proposed 2D growth mechanism. As a stronger field ligand compared with H_2_O, NH_3_ could bond with solvated Ni^2+^ and form [Ni(NH_3_)_6_]^2+^ (Fig. 2B I), competing with [Ni(H_2_O)_6_]^2+^ at the nuclei edge. However, unlike [Ni(H_2_O)_6_]^2+^, the addition of [Ni(NH_3_)_6_]^2+^ onto nuclei will terminate the expansion of 2D crystals since the edge NH_3_ hardly can deprotonate to maintain the localized negative charge (Fig. 2B V). Therefore, the resulting crystals would have smaller domain size, which is also observed from our experiment results.

Figure 2C shows the optical image of the obtained 2D Ni(OH)_2_ without additional NH_4_OH, where numerous 2D circular flakes are observed. And with 0.6 ml additional NH_4_OH (Fig. 2D), domain size of the flakes significantly decreases. Optical images of obtained 2D Ni(OH)_2_ with different amounts of additional NH_4_OH can be found in Fig. S7, showing with increasing amount of additional NH_4_OH, the domain size decreases and eventually bulk aggregates are yielded instead of 2D crystals. This is due to the hydrolysis of NH_4_OH, which brings more NH_3_ into the system to form more [Ni(NH_3_)_6_]^2+^ or monohydrate substituted [Ni(H_2_O)_x_(NH_3_)_6-x_]^2+^ (where x ≥ 1), which prevents the 2D expansion based on our proposed mechanism described above. This hypothesis is further supported by the UV–Vis measurements (Fig. 2E). Three absorption bands assigned left to right as ^3^A_2g_ → ^3^T_1g_ (^3^F), ^3^A_2g_ → ^3^T_1g_ (^3^F), ^3^A_2g_ → ^3^T_2g_, are consistent with homoleptic six-coordinate Ni(II) complexes^52^. The increased absorption intensity (purple curve) and blue color (Fig. 2E inset) with 0.6 ml additional NH_4_OH both suggest more [Ni(H_2_O)_x_(NH_3_)_6-x_]^2+^ are formed.

### Structural and crystallographic characterization of 2D α-Ni(OH)_2_

**Figure 3:**
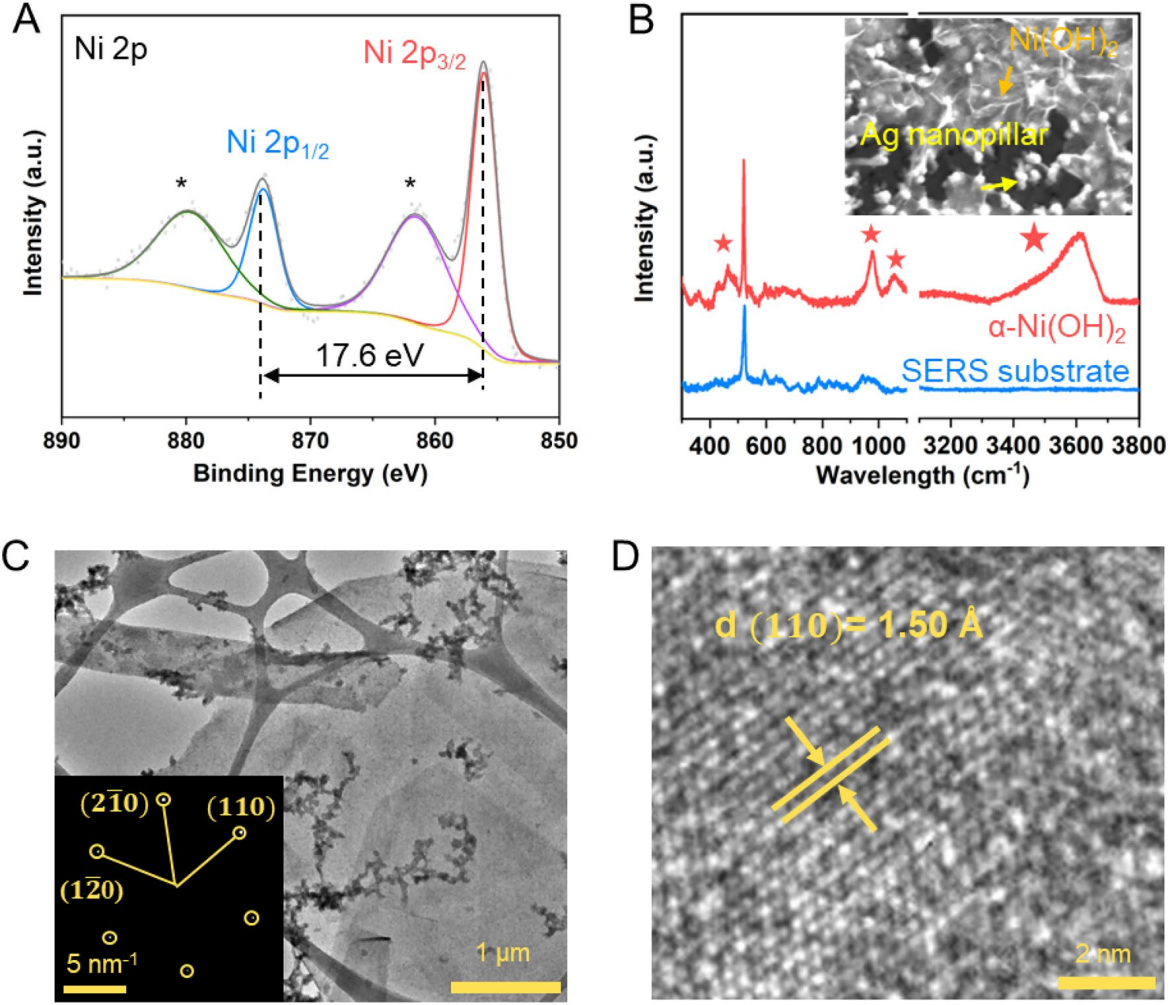
Crystallographic characterization of Ni(OH)_2_. (**A**) XPS spectra of Ni 2p, suggesting a 17.6 eV spin energy separation, which is characteristic of the Ni^2+^ in Ni(OH)_2_. (**B**) Raman spectra of Ag nanopillar SERS substrate with and without Ni(OH)_2_ thin flakes on. Characteristic peaks of α-Ni(OH)_2_ indicated by “stars” are observed and a fingerprint one is observed at 3610 cm^−1^. Inset: SEM image of Ag nanopillar SERS substrate with and without Ni(OH)_2_ thin flakes on. (**C**) Low-magnification TEM image. Inset: the SAED pattern. (**D**) High-magnification TEM image, the d-spacing of $\left(110\right)$ lattice plane is measured as 1.50 Å.

We further characterize the synthesized Ni(OH)_2_ flakes using XPS. The survey spectrum (Fig. S8A) clearly indicates the presence of Ni, O elements^53^ and some other elements from substrate and environment with low intensity. The core-level spectrum of Ni 2p (Fig. 3A) shows the two major peaks at 856.1 (Ni 2p_3/2_) and 873.8 eV (Ni 2p_1/2_) with a spin-orbital coupling caused splitting of 17.6 eV, which is characteristic of the Ni^2+^ ion in Ni(OH)_2_^54,55^. Meanwhile, the O1 s spectrum (Fig. S8B) is fitted with three peaks located at 531.1, 532.4 and 533.2 eV, representing Ni–O–H, O–Si–O and H–O–H, respectively^56^. The appearance of H–O-H signal suggests the existence of H_2_O in the crystal, mainly as intercalation species between layers to form α-Ni(OH)_2_.

To better probe the crystal phase of the 2D thin flakes, surface-enhanced Raman spectroscopy (SERS) measurement using a commercial Ag-nanopillar-coated substrate (Fig. 3B inset) is performed, as the synthesized α-Ni(OH)_2_ flakes are too thin to exhibit visible Raman signals using the normal Raman spectroscopy measurements. The fingerprint for identifying α-Ni(OH)_2_ from β-Ni(OH)_2_ or the mixture is observed at 3610 cm^−1^ (Fig. 3B)^12,57^. Moreover, the phonon mode of α-Ni(OH)_2_ is observed at 460 cm^−1^, the 2nd order phonon modes of α-Ni(OH)_2_ are observed at 979 and 1055 cm^−1^, further proving α phase of the synthesized Ni(OH)_2_.

More importantly, transmission electron microscopy (TEM) measurements are carried out to further reveal the structure in the flakes. The low-resolution TEM image in Fig. 3C reveals the 2D nature of a micrometer large domain of a typical sample. The selected area electron diffraction (SAED) (Fig. 3C inset) reveals the hexagonal lattice structure, matching well with the simulated lattice pattern (Fig. S9A)^58^. The sharp signals of the SAED patterns collected from multiple area suggest the high crystallinity of the sample crystal (Fig. S9B–D). Moreover, atomic-resolution TEM image (Fig. 3D) shows the spacing between $(110)$ planes is 1.50 Å.

Furthermore, the thermal stability of the materials and chemical stability in solvents are studied and showed in Fig. S10. In Fig. S10A, XPS spectra of Ni(OH)_2_ and NiO that oxidized under different temperatures are shown. The energy separation on Ni 2p spectra remains the same when the temperature is 150 °C and changes dramatically when the temperature is lifted to 160 °C, suggesting the synthesized 2D Ni(OH)_2_ is stable and does not decompose until 160 °C. Besides, the stability of the synthesized 2D Ni(OH)_2_ in different solvents, i.e., isopropyl alcohol (IPA), acetone, ethanol, toluene, are investigated. After 30 min of immersion in each solvent, 2D Ni(OH)_2_ flakes contain the same shape as pretreatment (Fig. S10B), which suggest its great chemical stability in organic solvents. The above stability tests provide important guidance for following device fabrication.

### Optical and electric characterization of 2D α-Ni(OH)_2_

**Figure 4:**
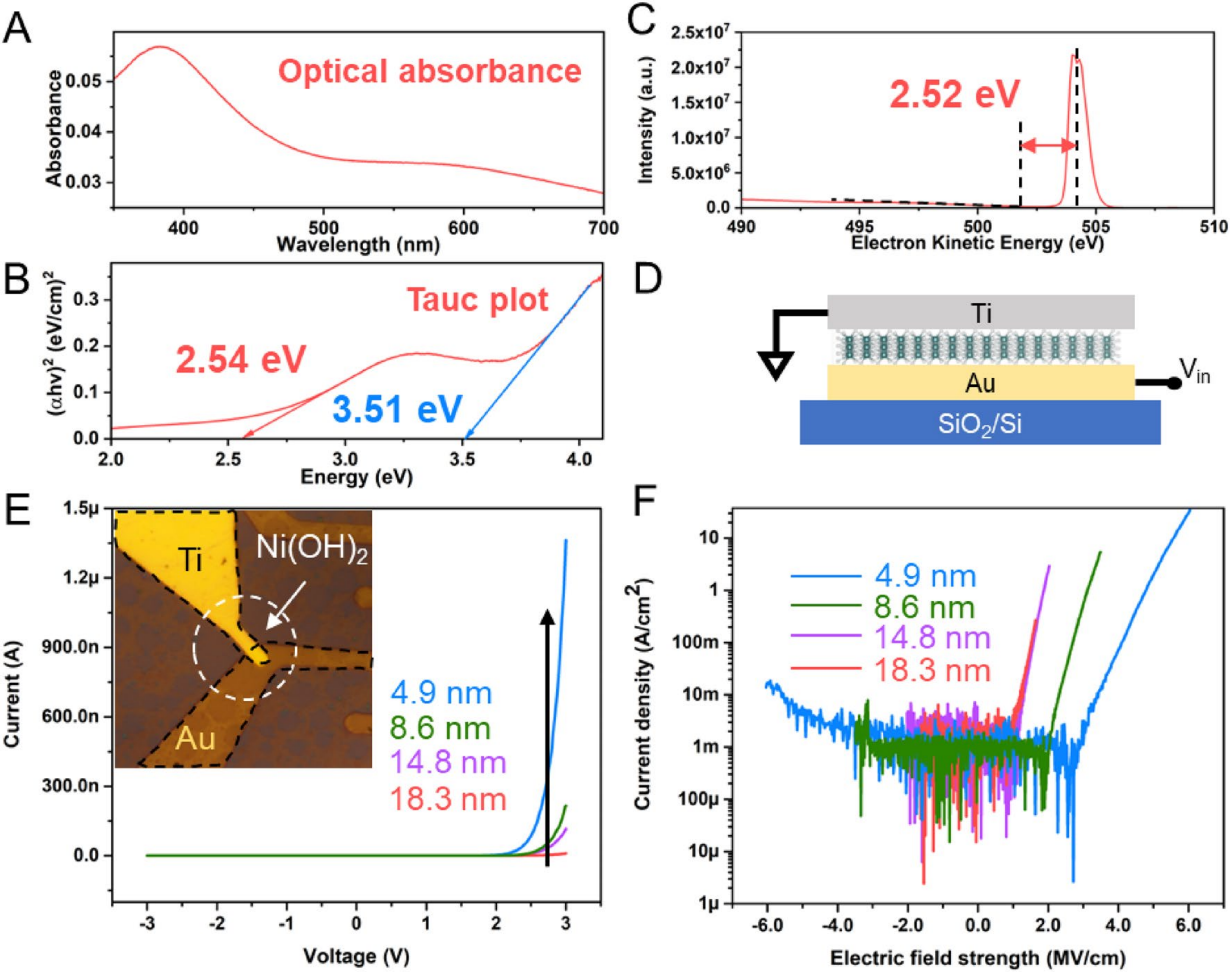
Optical and electrical characterizations of Ni(OH)_2_. (**A**) Optical absorption spectrum of few layers α-Ni(OH)_2_ thin flakes on ITO glass, measured by UV–Vis from 300 to 700 nm. (**B**) Tauc plot derived from (**A**), the extrapolated bandgaps are 2.54 eV and 3.51 eV, corresponding to few layers and bulk α-Ni(OH)_2_, respectively. (**C**) Bandgap extrapolated from reflected electrons energy loss spectroscopy (REELS) by measuring the gap between the center of elastic scattering peak and the cutoff of the low energy loss peak is 2.52 eV. (**D**) Schematic of Au-Ni(OH)_2_-Ti sandwich-like device. The top Ti electrode is grounded and voltage is applied on the bottom Au electrode for all measurements. (**E**) Typical I-V characteristics of samples with different thicknesses, from 4.9 to 18.3 nm. Current increases exponentially under positive bias on all four measured samples. Inset: optical image of the device. (**F**) Current density–electric field strength correlation of the tested samples with current in logarithm scale. The thinnest sample, 4.9 nm, exhibits the highest breakdown field, 4.47 MV/cm.

Ultraviolet–visible spectroscopy (UV–Vis) is carried out on Ni(OH)_2_ thin flakes spin-coated on indium tin oxide (ITO) coated glass. The optical absorption spectrum (Fig. 4A) is obtained from 300 to 700 nm. In addition, Tauc plot (Fig. 4B) is derived on the direct bandgap^33,59^ and two bandgaps are extrapolated, 2.54 eV and 3.51 eV, corresponding to few-layers and bulk Ni(OH)_2_, respectively^59,60^. Note that the bandgap of 2D α-Ni(OH)_2_ is smaller than that of its bulk counterpart, which has been reported as an exceptional deviation from the quantum confinement effect^59^ and is opposite from most other 2D materials^61,62^. Moreover, the electronic bandgap is also measured using reflected electrons energy loss spectroscopy (REELS). As shown in Fig. 4C, a bandgap of 2.52 eV is obtained by measuring the distance between the center of elastic scattering peak and the cutoff of the low energy loss region.

Together with its large domain size, the relatively large bandgap of 2D α-Ni(OH)_2_ motivates us to investigate its electric properties. An Au-Ni(OH)_2_-Ti sandwich-like structure is fabricated for electric property measurements (Fig. 4D), where the top Ti electrode is grounded and voltage is applied on the bottom Au electrodes. Figure 4E shows the typical I-V curves of the devices (as shown in Fig. 4E inset) based on samples with different thicknesses from 4.9 to 18.3 nm. For all four devices, the current grows exponentially with positive voltage, while a much lower or negligible current is observed with negative voltage. This unipolar conducting characteristic is attributed to the difference in Schottky barrier height (S.B.H.) at the Au-Ni(OH)_2_ and Ti-Ni(OH)_2_ interfaces. Given a much higher work function of Au compared to Ti, Au is expected to form a higher barrier with 2D Ni(OH)_2_^63^. When a positive voltage is applied on the Au electrode, this barrier is forward biased, resulting in an exponential I-V curve once the voltage exceeds its threshold value (Fig. S11A). Otherwise, the barrier is reversely biased, and no current is present before breakdown. We further convert the bias voltage into electric field strength to extract the breakdown field of each device (Fig. 4F), which is 4.47, 2.92, 1.73 and 1.64 MV/cm for 4.9, 8.6, 14.8, and 18.3 nm Ni(OH)_2_ flakes, respectively (Fig. S11B). The reduced electric strength at higher thickness may come from the higher density of defects at higher thickness, which is also observed in other 2D WBG materials (e.g., h-BN, SiO_2_^64^. Nonetheless, the large bandgap and high breakdown strength suggest its great potential for high voltage operation compared to SiC and GaN (Fig. S11C). Thus, the addition of Ni(OH)_2_ to the 2D family provides a promising candidate as WBG semiconductor for future micro-nanoelectronics devices. From the synthesis point of view, the results demonstrate the great continuity and uniformity of the synthesized 2D Ni(OH)_2_ flakes.

## Conclusion

In conclusion, we achieve the largest ever reported 2D Ni(OH)_2_ (> 20 μm) and Co(OH)_2_ (> 40 μm) crystals down to a few nanometers in the hydrothermal synthesis process with a comprehensive understanding of the growth mechanism. Specifically, a direct relationship between spin-state controlled metal–ligand geometry and macroscopic morphology of the LTMHs is built, illustrating the preferred geometry from the growth. A 2D growth mechanism via edge-on condensation at atomic level is established for the hydrothermal synthesis of Ni(OH)_2_ through a systematic investigation on the influence of key parameters. The reaction-morphology relationship elaborated herein provides a blueprint for a well-guided approach towards the successful design and development of more 2D LTMHs. Moreover, the detailed characterization on the synthesized Ni(OH)_2_ flakes shows the high crystallinity, α phase, and large bandgap of 2.5 eV. Electrical measurements further suggest its great potential of as a WBG semiconductor for high voltage operation in 2D electronic devices. Considering the great scalability of the hydrothermal synthesis method, our work suggests a promising future of joining LTMHs in the 2D family as a group of important members for future micro-nanoelectronics.

## Methods

### Hydrothermal synthesis

The synthesis is initiated by combining 0.504 g nickel nitrate hexahydrate, an inexpensive, commercially available salt, with 0.456 g carbamide, commonly known as urea, in the mixture of ethylene glycol (28 ml) and deionized water (4 ml). After 30 min stirring under room temperature, the solution is transferred to a 100 ml Teflon vessel, which is sealed into a hydrothermal autoclave reactor afterwards. For an optimal synthesis, the reactor is sent into an oven, in which the temperature is raised up to 120 °C at a speed of 1.5 °C/min. After 18 h soaking, the reactor is cooled down to room temperature as the same speed as heating up, then the solution is washed by ethanol and deionized (DI) water for three times each and eventually dispersed in DI water (usually ~ 40–50 ml) for storage. The obtained flakes are high yielding on gram scales and gradually sinking to bottom over time.

### Sample preparation

The synthesized thin Ni(OH)_2_ flakes are extracted from the bulk material by centrifugation. After 4 h centrifuge at 9500 rpm, 3–4 drops (~ 0.15–0.2 ml) of clear supernatant is drop-casted on 1 × 1 cm: (1) 300 nm SiO_2_/Si substrates for optical images, AFM, SEM, (2) Si substrates for XPS, (3) copper grids and silicon nitrite grids for TEM, (4) Ag-nanopillar-coated substrate for SERS and dried by heating on hot plate at 80 °C for 2 h. The clear supernatant and dispersion are spin-coated on ITO glasses for UV–Vis measurements. The sediment is collected and dried at 80 °C for 12 h for PXRD.

### Chemicals and materials

All reagents (carbamide with purity 98 + %, nickel nitrate hexahydrate with purity 98%) are purchased from Fisher chemical and used without further purification, with the exception of nickel nitrate hexahydrate, which is recrystallized from supersaturated aqueous solution.

### Characterization techniques

Optical images are collected by optical microscopy (Nikon DS-Ri2). AFM topography is acquired on a Bruker Dimension 3000 in a tapping mode. SEM images are taken on Zeiss Supra 55. XPS spectra are collected from PHI Versaprobe II. TEM measurements are performed on a FEI Tecnai Osiris TEM, operating at a 200 keV accelerating voltage. SAED is measured on a JEOL 2100 TEM. The SAED simulation is performed through STEM_CELL software. PXRD scanning are performed on a Bruker D2 Phaser with Cu Kα radiation of wavelength λ = 1.54184 Å at 30 kV and 10 mA. SERS measurement is performed on a Horiba-JY T64000, using a triple-grating mode with 1800 g mm^–1^ gratings, and a 532 nm laser line. Solution- and solid-state UV–Vis spectra are taken with Agilent Cary 5000. REELS measurement is carried out on Thermo Scientific Nexsa G2 and data is analyzed using Avantage software.

### Electric measurements

The Au electrodes are firstly fabricated on the SiO_2_/Si surface, 2D Ni(OH)_2_ flakes are then transferred on top of the Au electrode. Ti electrode is then deposited on the top of 2D Ni(OH)_2_ form an Au-Ni(OH)_2_-Ti sandwich-like structure from bottom to top. During the measurement, the top Ti electrode is grounded and voltage is applied on the bottom Au electrodes.

### Supplementary Information

Supplementary Information.

## Data Availability

All data generated or analyzed during this study are included in this published article and its supplementary information file.

## References

[1] Tan J, Li S, Liu B, Cheng H-M (2021). Structure, preparation, and applications of 2D material-based metal-semiconductor heterostructures. Small Struct..

[2] K. S. Novoselov, A. K. Geim, S. V. Morozov, D. Jiang, Y. Zhang, S. V. Dubonos, I. V. G. and A. A. F. Electric field effect in atomically thin carbon films. *Science.* **306**, 666–669 (2016). 10.1126/science.1102896 15499015

[3] Dutta R (2023). Optical enhancement of indirect bandgap 2D transition metal dichalcogenides for multi-functional optoelectronic sensors. Adv. Mater..

[4] Ye F, Liu Q, Xu B, Feng PXL, Zhang X (2023). Ultra-high interfacial thermal conductance via double hBN encapsulation for efficient thermal management of 2D electronics. Small.

[5] Grudinin DV (2023). Hexagonal boron nitride nanophotonics: a record-breaking material for the ultraviolet and visible spectral ranges. Mater. Horizons.

[6] Cheon G (2017). Data mining for new two- and one-dimensional weakly bonded solids and lattice-commensurate heterostructures. Nano Lett..

[7] Novoselov, K. S., Mishchenko, A., Carvalho, A. & Castro Neto, A. H. 2D materials and van der Waals heterostructures. *Science* **353**, (2016). 10.1126/science.aac9439 27471306

[8] Feng K, Li W, Xie S, Lu X (2014). Nickel hydroxide decorated hydrogenated zinc oxide nanorod arrays with enhanced photoelectrochemical performance. Electrochim. Acta.

[9] Camardese J, McCalla E, Abarbanel DW, Dahn JR (2014). Determination of shell thickness of spherical core-shell Ni x Mn 1–x (OH) 2 particles via absorption calculations of X-ray diffraction patterns. J. Electrochem. Soc..

[10] Tizfahm J (2014). Supercapacitive behavior of β-Ni(OH)2 nanospheres prepared by a facile electrochemical method. Colloids Surf. A Physicochem. Eng. Asp..

[11] Jayashree, R. S. & Vishnu Kamath, P. Factors governing the electrochemical synthesis of α-nickel (II) hydroxide. *J. Appl. Electrochem.* **29**, 449–454 (1999).

[12] Hall DS, Lockwood DJ, Poirier S, Bock C, MacDougall BR (2014). Applications of in situ Raman spectroscopy for identifying nickel hydroxide materials and surface layers during chemical aging. ACS Appl. Mater. Interfaces.

[13] Cui, H., Xue, J., Ren, W. & Wang, M. Ultra-high specific capacitance of β-Ni(OH)2monolayer nanosheets synthesized by an exfoliation-free sol-gel route. *J. Nanoparticle Res.* **16**, (2014).

[14] Ma X, Liu J, Liang C, Gong X, Che R (2014). A facile phase transformation method for the preparation of 3D flower-like β-Ni(OH)2/GO/CNTs composite with excellent supercapacitor performance. J. Mater. Chem. A.

[15] Wang M, Ni Y, Cao L, Zhao D, Ma X (2013). Porous Ni/β-Ni(OH)2 superstructures: Rapid solvothermal synthesis, characterization, and electrochemical property. J. Colloid Interface Sci..

[16] Harvey A (2016). Production of Ni(OH)2 nanosheets by liquid phase exfoliation: From optical properties to electrochemical applications. J. Mater. Chem. A.

[17] Acharya P (2022). Fe coordination environment, Fe-incorporated Ni(OH)2Phase, and metallic core are key structural components to active and stable nanoparticle catalysts for the oxygen evolution reaction. ACS Catal..

[18] Li Y, Tong R, Zhang W, Peng S (2022). Pre-intercalation of phosphate into Ni(OH)2/NiOOH for efficient and stable electrocatalytic oxygen evolution reaction. J. Catal..

[19] Sun W, Rui X, Ulaganathan M, Madhavi S, Yan Q (2015). Few-layered Ni(OH)2 nanosheets for high-performance supercapacitors. J. Power Sources.

[20] Aghazadeh M, Dalvand S, Hosseinifard M (2014). Facile electrochemical synthesis of uniform β-Co(OH)2 nanoplates for high performance supercapacitors. Ceram. Int..

[21] Yu J, Hai Y, Cheng B (2011). Enhanced photocatalytic H2-production activity of TiO 2 by Ni(OH)2 cluster modification. J. Phys. Chem. C.

[22] Deng J, Wu F, Gao S, Dionysiou DD, Huang L-Z (2022). Self-activated Ni(OH)2 cathode for complete electrochemical reduction of trichloroethylene to ethane in low-conductivity groundwater. Appl. Catal. B Environ..

[23] Patil, S. J. *et al.* Fluorine engineered self-supported ultrathin 2D nickel hydroxide nanosheets as highly robust and stable bifunctional electrocatalysts for oxygen evolution and urea oxidation reactions. *Small* **18**, (2022). 10.1002/smll.202103326 34889512

[24] Huang ZH (2021). An electro-activated bimetallic zinc-nickel hydroxide cathode for supercapacitor with super-long 140,000 cycle durability. Nano Energy.

[25] Wu Y (2021). Rational design of cobalt–nickel double hydroxides for flexible asymmetric supercapacitor with improved electrochemical performance. J. Colloid Interface Sci..

[26] Wan L, Wang P (2021). Recent progress on self-supported two-dimensional transition metal hydroxides nanosheets for electrochemical energy storage and conversion. Int. J. Hydrogen Energy.

[27] Dong L, Chu Y, Sun W (2008). Controllable synthesis of nickel hydroxide and porous nickel oxide nanostructures with different morphologies. Chem. - A Eur. J..

[28] Wang Y, Gai S, Niu N, He F, Yang P (2013). Fabrication and electrochemical performance of 3D hierarchical β-Ni(OH)2 hollow microspheres wrapped in reduced graphene oxide. J. Mater. Chem. A.

[29] Mai HD, Kim S, Yoo H (2020). Gold nanodots-decorated nickel hydroxide nanoflowers for enhanced electrochemical oxygen evolution activity. J. Ind. Eng. Chem..

[30] Gao H (2022). Phase-controllable synthesis of ultrathin molybdenum nitride crystals via atomic substitution of MoS2. Chem. Mater..

[31] Cao J (2020). Realization of 2D crystalline metal nitrides via selective atomic substitution. Sci. Adv..

[32] Tang, Z., Tong, C., Geng, W., Zhang, D. & Liu, L. Two-dimensional Ni ( OH ) 2 -XS 2 ( X = Mo and W ) heterostructures. *2D Mater*. **2**, (2015).

[33] Tang ZK, Liu WW, Zhang DY, Lau WM, Liu LM (2015). Tunable band gap and magnetism of the two-dimensional nickel hydroxide. RSC Adv..

[34] Wei XL, Tang ZK, Guo GC, Ma S, Liu LM (2015). Electronic and magnetism properties of two-dimensional stacked nickel hydroxides and nitrides. Sci. Rep..

[35] Hall, D. S., Lockwood, D. J., Bock, C. & MacDougall, B. R. Nickel hydroxides and related materials: A review of their structures, synthesis and properties. *Proc. R. Soc. A Math. Phys. Eng. Sci.* **471**, (2015). 10.1098/rspa.2014.0792 PMC4309132 25663812

[36] Yang LX (2007). Hydrothermal synthesis of nickel hydroxide nanostructures in mixed solvents of water and alcohol. J. Solid State Chem..

[37] Zaman, M. B., Poolla, R., Khandy, S. A., Modi, A. & Tiwari, R. K. Thioglycolic acid assisted hydrothermal growth of SnS 2D nanosheets as catalysts for photodegradation of industrial dyes. *Nanotechnology* **32**, (2021). 10.1088/1361-6528/abec09 33662944

[38] Wu M (2021). Uniform rotate hydrothermal synthesis of V6O13 nanosheets as cathode material for lithium-ion battery. J. Alloys Compd..

[39] Liu H (2020). Nickel manganese hydroxides with thin-layer nanosheets and multivalences for high-performance supercapacitor. Res. Phys..

[40] Deng, Y. feng *et al.* One-step synthesis of 2D vertically-aligned hybrid CuSe@NiSe nanosheets for high performance flexible supercapacitors. *J. Alloys Compd.* **892**, 162159 (2022).

[41] Jahn HA, Teller E (1937). Stability of Polyatom ic M olecules in D egenerate E lectronic States I-O rbital D egeneracy. Proc. R. Soc. London.

[42] Bersuker IB (2001). Modern aspects of the Jahn-Teller effect theory and applications to molecular problems. Chem. Rev..

[43] Bersuker IB (2021). Jahn-Teller and Pseudo-Jahn-Teller effects: From particular features to general tools in exploring molecular and solid state properties. Chem. Rev..

[44] Li J, Li Z, Zhan F, Shao M (2021). Phase engineering of cobalt hydroxide toward cation intercalation. Chem. Sci..

[45] Rovetta, A. A. S. *et al.* Cobalt hydroxide nanoflakes and their application as supercapacitors and oxygen evolution catalysts. *Nanotechnology* **28**, (2017). 10.1088/1361-6528/aa7f1b 28696333

[46] Balasubramanian, P., He, S. Bin, Deng, H. H., Peng, H. P. & Chen, W. Defects engineered 2D ultrathin cobalt hydroxide nanosheets as highly efficient electrocatalyst for non-enzymatic electrochemical sensing of glucose and L-cysteine. *Sensors Actuators, B Chem.* **320**, 128374 (2020).

[47] Cotton FA, Daniels LM, Murillo CA, Quesada JF (1993). Hexaaqua dipositive ions of the first transition series: New and accurate structures expected and unexpected trends. Inorg. Chem..

[48] Halcrow MA (2013). Jahn-Teller distortions in transition metal compounds, and their importance in functional molecular and inorganic materials. Chem. Soc. Rev..

[49] Chen J, Xu J, Zhou S, Zhao N, Wong CP (2015). Facile and scalable fabrication of three-dimensional Cu(OH)2 nanoporous nanorods for solid-state supercapacitors. J. Mater. Chem. A.

[50] Satpathy BK, Patnaik S, Pradhan D (2022). Room-Temperature Growth of Co(OH)2Nanosheets on Nanobelt-like Cu(OH)2Arrays for a binder-free high-performance all-solid-state supercapacitor. ACS Appl. Energy Mater..

[51] De Soler-Illia GJAA (1999). Synthesis of nickel hydroxide by homogeneous alkalinisation. Precipitation mechanism. Chem. Mater..

[52] Stankus, T. *Inorganic chemistry*. *Serials Librarian* vol. 27 (1996).

[53] Tian J (2019). 2D nanoporous Ni(OH)2 film as an electrode material for high-performance energy storage devices. RSC Adv..

[54] Cai X (2015). Solvothermal synthesis of NiCo-layered double hydroxide nanosheets decorated on RGO sheets for high performance supercapacitor. Chem. Eng. J..

[55] He F (2014). In situ fabrication of nickel aluminum-layered double hydroxide nanosheets/hollow carbon nanofibers composite as a novel electrode material for supercapacitors. J. Power Sources.

[56] Rani JR, Thangavel R, Kim M, Lee YS, Jang JH (2020). Ultra-high energy density hybrid supercapacitors using mno2/reduced graphene oxide hybrid nanoscrolls. Nanomaterials.

[57] Hall DS, Lockwood DJ, Poirier S, Bock C, MacDougall BR (2012). Raman and infrared spectroscopy of α and β phases of thin nickel hydroxide films electrochemically formed on nickel. J. Phys. Chem. A.

[58] Mafakheri, E. *et al.* Realization of electron vortices with large orbital angular momentum using miniature holograms fabricated by electron beam lithography. *Appl. Phys. Lett.* **110**, (2017).

[59] Nagli, M. & Caspary Toroker, M. Communication: Nickel hydroxide as an exceptional deviation from the quantum size effect. *J. Chem. Phys.* **149**, (2018). 10.1063/1.5051202 30316282

[60] Pareek A, Paik P, Borse PH (2016). Stable hydrogen generation from Ni- and Co-based co-catalysts in supported CdS PEC cell. Dalt. Trans..

[61] Chaves, A. *et al.* Bandgap engineering of two-dimensional semiconductor materials. *Npj 2D Mater. Appl.* **4**, (2020).

[62] Jiang T (2014). Valley and band structure engineering of folded MoS 2 bilayers. Nat. Nanotechnol..

[63] Kim C (2017). Fermi level pinning at electrical metal contacts of monolayer molybdenum dichalcogenides. ACS Nano.

[64] Ranjan A (2021). Dielectric breakdown in single-crystal hexagonal boron nitride. ACS Appl. Electron. Mater..

