# Supplementary material for: Synthesis of 2D layered transition metal (Ni, Co) hydroxides via edge-on condensation
Source: Sci Rep. 2024 Feb 15;14:3817. doi: 10.1038/s41598-024-53969-2 (PMC10869340; doi:10.1038/s41598-024-53969-2)
Supplement: Supplementary file 1 — Supplementary Information. [file 41598_2024_53969_MOESM1_ESM.pdf]

Supplementary Information

Synthesis of 2D Layered Transition Metal (Ni, Co) Hydroxides via Edge-on Condensation

Lu Ping[1*], Gillian E. Minarik[2*], Hongze Gao[2], Jun Cao[2], Tianshu Li[1], Hikari Kitadai[2], Xi Ling[1,2,3†]

[1] Division of Materials Science and Engineering, Boston University, 15 St. Mary's Street, Boston, MA 02215, USA.

[2] Department of Chemistry, Boston University, 590 Commonwealth Avenue, Boston, MA 02215, USA.

[3] The Photonics Center, Boston University, 8 St. Mary's Street, Boston, MA 02215, USA.

*These authors contributed equally to this work.

[†]Corresponding author. Email: xiling@bu.edu

This document contains:

Section 1. Testification of 2D growth mechanism through key parameter tuning of α-$Ni(OH)_2$ synthesis

Section 2. Supplementary figures

Section 1. Testification of 2D growth mechanism through key parameter tuning of α-$Ni(OH)_2$ synthesis

In order to maximize the 2D domains, the rate of crystal growth over nucleation first needs to be amplified. With slower nucleation, the Ni^{2+} ions stay dissolved as $[Ni(H_2O)_6]^{2+}$ units without prematurely crashing out as polycrystalline or amorphous solid with bulk morphology. In an ideal case where nucleation is minimized, promoting growth in the 2D plane is the next target. With the above understanding, our strategy of synthesizing large 2D LTMHs emphasizes promoting *ab* in-plane isotropy, which involves systematic control of parameters including cooling rate, soaking temperature, starting pH, as detailed in the following.

We first investigate the cooling rate on the morphology of the $Ni(OH)_2$. We performed the synthesis at 120 °C under the cooling rate of 0.5, 1.5 and 3.0 °C/min. At 3.0 °C/min. We observed many random shaped flakes with rough surfaces (Fig. S12 A), on which islands and aggregates formed. As the cooling rate decreases to 1.5 °C/min (Fig. S12 B), circular thin flakes are easily found with average domain size of ~20 μm, which is an exciting new record for 2D LTMHs synthesis. Interestingly, when the cooling is further slowed down to 0.5 °C/min (Fig. S12 C), the average domain size of obtained flakes is significantly smaller (around 1-2 μm) and thicker. The reason is that when cooling rate is decreased, the precipitation of seed crystals is largely slowed down and crystal growth is significantly encouraged. Thus, edge-on condensation happens rapidly on very limited amounts of seed crystals, overcoming the surface saturation of OH^- and leading to a disordered interlayer add-on, eventually built 3D crystals and limited the size on intra-planar dimension. Moreover, PXRD results show no distinguishable difference among the $Ni(OH)_2$ synthesized from different cooling rates (Fig. S12 D), suggesting cooling rate only subtly changed domain size and surface morphology without altering the crystal structure.

We further investigate the effect of the pH on the synthesis, since $Ni(OH)_2$ is reported preferably formed and stable in the pH range of 9-13.[1] The sealed autoclave vessel used in the hydrothermal synthesis prevents the *in situ* monitoring of pH over the reaction, thus, we only control the initial pH of the solution and evaluate trends in outcomes. The initial pH value is adjusted from 5.57 to 7.60 by adding potassium hydroxide (KOH) while the cooling rates is fixed at 1.5 °C/min and the reaction temperature at 120 °C. The optical images show that the optimal 2D morphology is produced when no KOH is added, which reliably corresponds to an initial pH of 5.57 (± 0.2) (Fig. S13 A). With raised pH, thin circular flakes decrease in size and abundance (Fig. S13 B). Until pH=7.60, only small $Ni(OH)_2$ particles arranged into amorphous films are observed (Fig. S13 C). Without extra KOH addition, the decomposition of urea provides gentle conditions in which OH^- anions are generated steadily, promoting crystal growth and reducing the chance of nucleation. In this way, the solvated $[Ni(H_2O)_6]^{2+}$ have more time to spend in solution and have more opportunity to incorporate to existing nuclei and crystals (Fig. 2A II), rather than rapidly precipitating as particles. In contrast, the extra addition of OH^- from KOH results in the supersaturation of $Ni(OH)_2$ in the solution and promotes fast precipitation of amorphous solid $Ni(OH)_2$ instead of intra-planar growth. Furthermore, the introduction of extra OH^- species slows down the urea hydrolysis, resulting in less production of ammonia, and consequently less promotable to form α phase, as ammonia has widely been identified as an intercalation species in $Ni(OH)_2$.[2] The PXRD clearly shows a transformation from mostly α-phase to multi-phase to β-phase $Ni(OH)_2$ when increasing the pH (Fig. S13 D). With the increase of the starting pH, PXRD peak at 2θ=12.5 ° from the d (001) of α-phase (grey arrow) vanishes and peak at 2θ=15.1 ° from the d (001) of β-phase (purple arrow) appears, along with the disappearance of α-(301) and emergence of β-(100) and β-(101) (green arrows) at 2θ=33.4 °, 38.2 °.[3,4] The increased integral intensity of (001) plane from β-

phase implies the presence of in-plane growth, however, smooth 2D morphology is not observed upon transition to the β-phase.

Another important factor we investigated is the soaking temperature. We perform the synthesis at various temperatures from 80 °C to 100 °C, 120 °C, 140 °C and 160 °C, where the cooling rate is set as a constant, 1.5 °C/min. At 80 °C (Fig. S14 A), only amorphous and bulky aggregates are observed, which persists when the temperature is elevated to 100 °C (Fig. S14 B). We believe that under low temperatures, the autogenous pressure generated in the fixed volume is too low to provide high supersaturation for $[Ni(H_2O)_6]^{2+}$ units to form and add onto the seed crystals. Accordingly, as the temperature increases to 120 °C (Fig. S14 C), large circular flakes are observed. However, at even higher temperatures (e.g., 140 °C, Fig. S14 D), surface defects, holes, and crimps are more widely observed in the optical image. This is due to the increased production of free ammonia from promoted urea hydrolysis. The excess ammonia forms $[Ni(NH_3)_6]^{2+}$ complexes, effectively consuming Ni^{2+} ions and preventing the formation of $[Ni(H_2O)_6]^{2+}$ units. Different from H_2O, the deprotonation hardly happens for NH_3, thus, the seed crystals lose attraction to $[Ni(H_2O)_6]^{2+}$ units, stopping the growth of the crystal. Notably, with the temperature further increasing to 160 °C (Fig. S14 E), the average size decreases to ~5 μm and some bulky aggregates are found gathering around the circular flakes, which is another consequence of nuclei edge blocking. The higher the temperature is, the more $[Ni(NH_3)_6]^{2+}$ and less $[Ni(H_2O)_6]^{2+}$ complexes are formed, then the more terminal groups on the existing nuclei and crystals are occupied by NH_3. Thus, edge substitution cannot proceed to achieve intra-planar growth, leading to smaller crystal size eventually.

PXRD spectra of samples synthesized under different temperatures is shown in Fig. S14 F. The peak from α-(100) at 18.3 ° (indicated by the green arrow) decreases significantly when increasing the temperature from 120 to 160 °C, suggesting intralayer growth along (100) direction is diminished. This result matches with the decreasing average domain size observed .

Moreover, as the temperature increased, the β (001) peak at 21.7 ° (grey arrow) becomes smaller and less visible, indicating the decreased proportion of β phase space in the α/β-interstratification structure, which is the consequence of interlayer swelling under high temperature, suggesting high temperature would produce purer and highly ordered α-$Ni(OH)_2$. Besides, we also observed a set of small peaks (purple arrow) along the 140 °C spectrum (Fig. S14 G) in the range of 30 to 80°, suggesting the disorder and rearrangement of the crystal structure due to an ongoing phase transition at this intermediate temperature.

In a nutshell, the comprehensive investigation of key parameters on the synthesis supports our hypothesis on the edge-on condensation 2D growth mechanism. Briefly, the synthesis starts with the formation of seed crystals and octahedral $[Ni(H_2O)_6]^{2+}$ units, followed by continuous water and hydroxyl ligand-substitution. We also realize that controlling the moderate nucleation and growth rate, together with an acidic environment are the key to promote the 2D intra-planar crystal growth of $Ni(OH)_2$.

Section 2. Supplementary figures

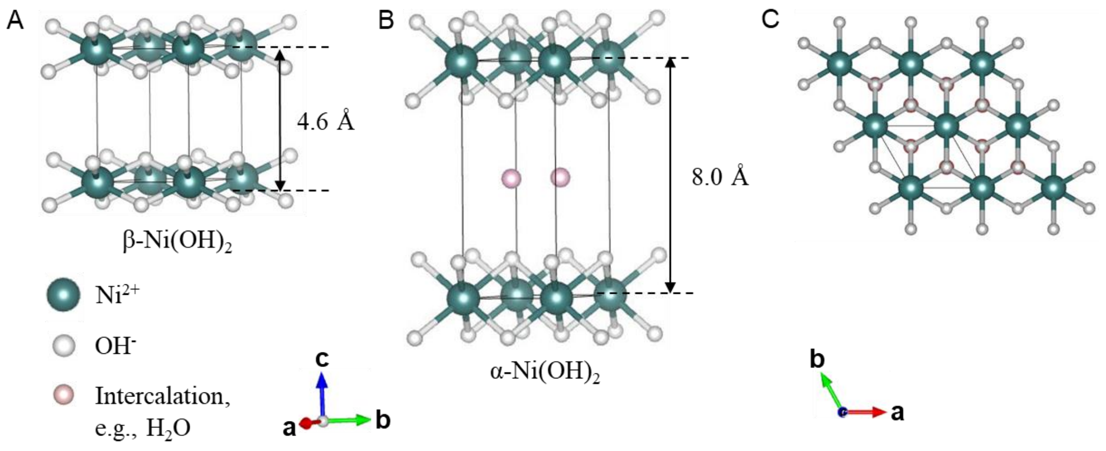

Fig. S1. Crystal structure of (**A**) β-Ni$(OH)_2$, (**B**) α-$(OH)_2$ from side view. Both have layered structure. The major difference is the interlayer distance due to intercalation, which is 4.6 Å and 8.0 Å for β and α phase, respectively.[1] (**C**) Shared hexagonal lattice of β-Ni$(OH)_2$ and α-$(OH)_2$ from top view.

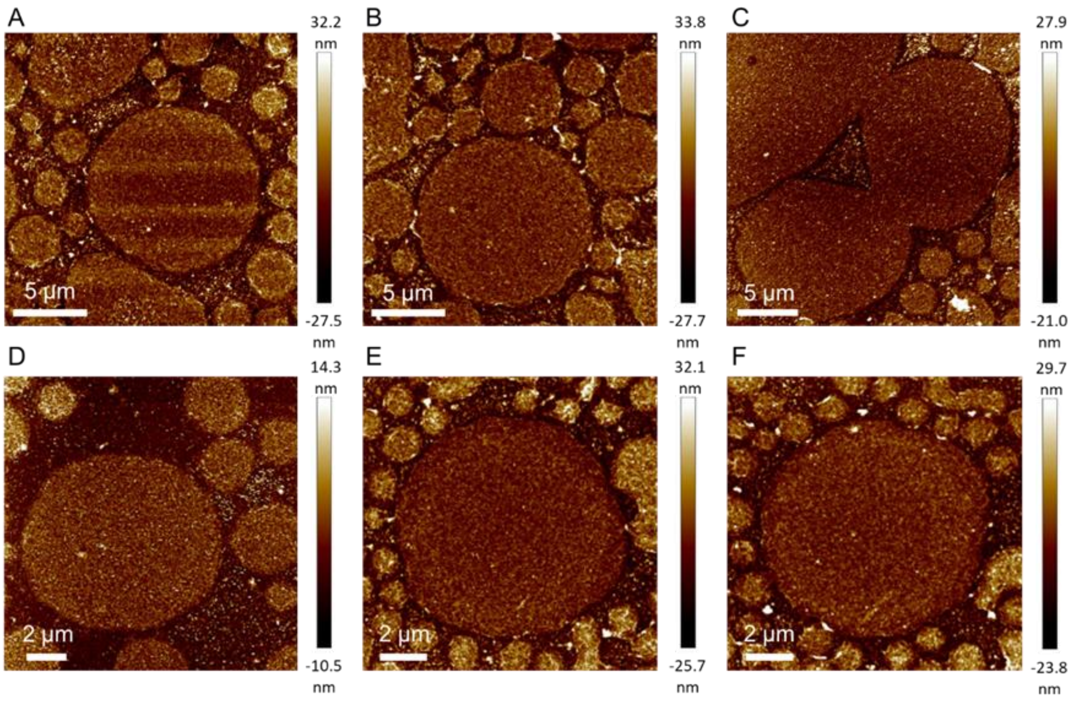

Fig. S2. AFM images of six 2D Ni$(OH)_2$ flakes (A-F), showing the continuous crystal domain in a large area (>10 µm).

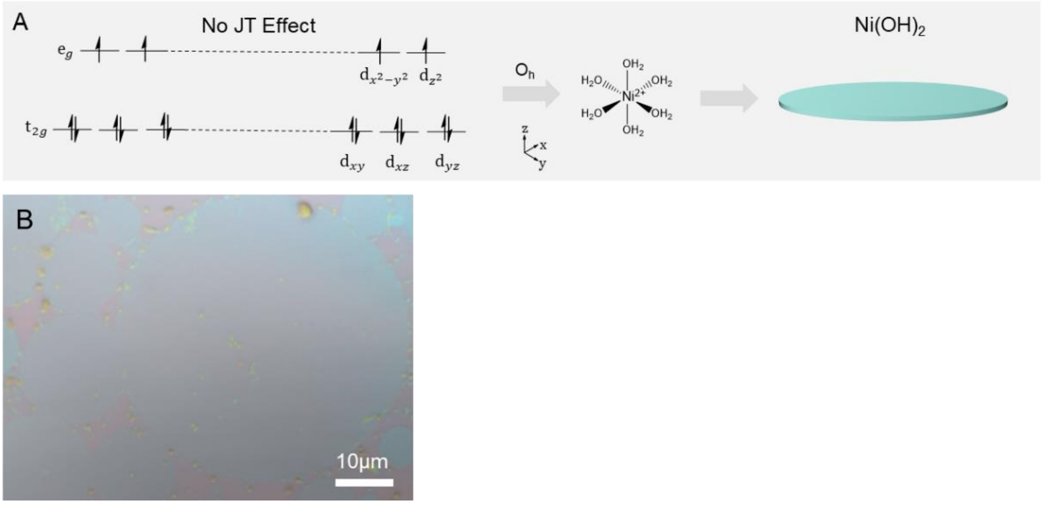

Fig. S3. Spin states directed geometry of Ni^{2+} and optical image of synthesized 2D $Ni(OH)_2$ crystals. (A) $[Ni(H_2O)_6]^{2+}$ has perfect O_h symmetry with identical bond length and electron density on all six Ni-H_2O metal-ligand coordination bonds, yielding abundant (quasi-) circular 2D flakes. (**B**) Optical image of synthesized 2D $Ni(OH)_2$ flakes on SiO_2/Si substrate, domain size reaches ~20 μm on one direction.

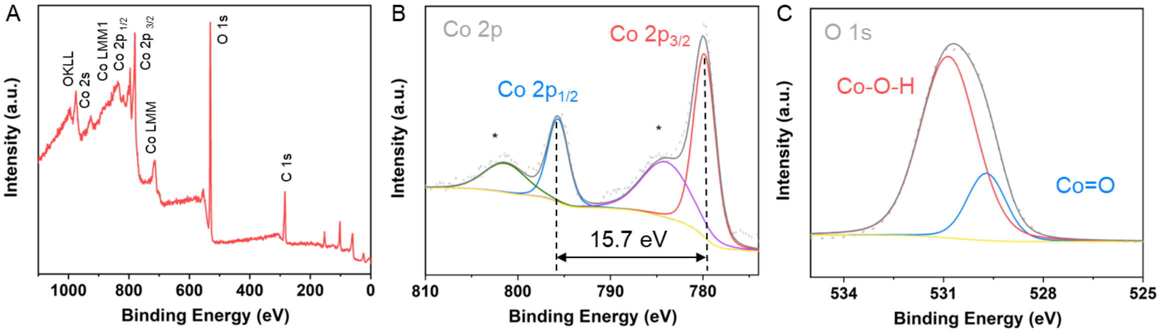

Fig. S4. Chemical states of synthesized 2D $Co(OH)_2$, detected by XPS. (**A**) Survey spectrum, (**B**) Co 2p spectrum, (**C**) O 1s spectrum of $Co(OH)_2$ thin flakes, indicating the occurrence of Co and O elements at their binding energies, with a characteristic spin energy separation of 15.7 eV. The slight amount of CoO might be generated during synthesis or oxidized from $Co(OH)_2$ during storage.

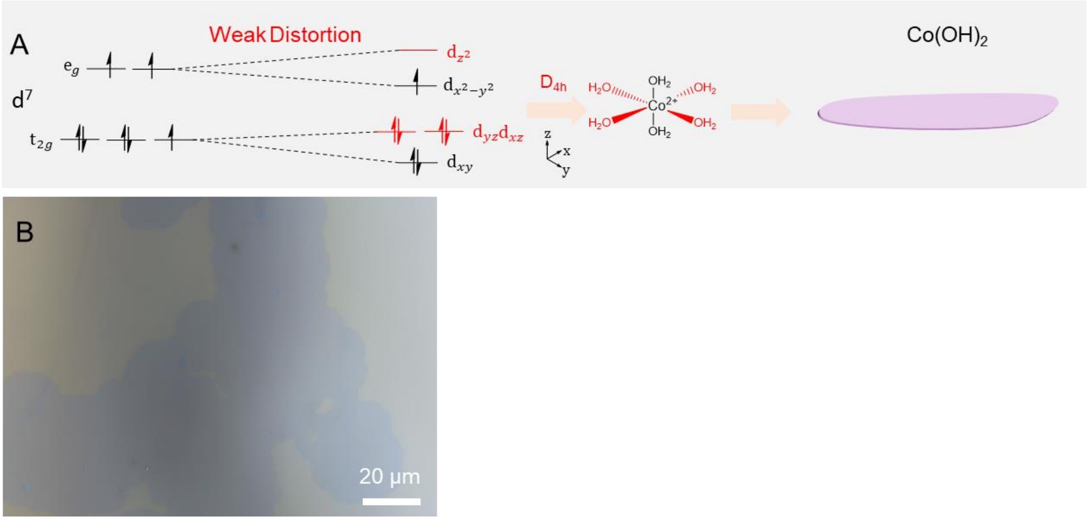

Fig. S5. Spin states directed geometry of $[Co(H_2O)_6]^{2+}$ metal-ligand complex and the optical image of synthesized 2D $Co(OH)_2$ crystals. (**A**) $[Co(H_2O)_6]^{2+}$ weakly distorts to D_{4h} symmetry with weak compression along the z-axis, yielding fewer observed 2D flakes with irregular shape and varying thickness. (**B**) Optical image of synthesized 2D $Co(OH)_2$ flakes on SiO_2/Si substrate, domain size reaches ~40 μm on one direction.

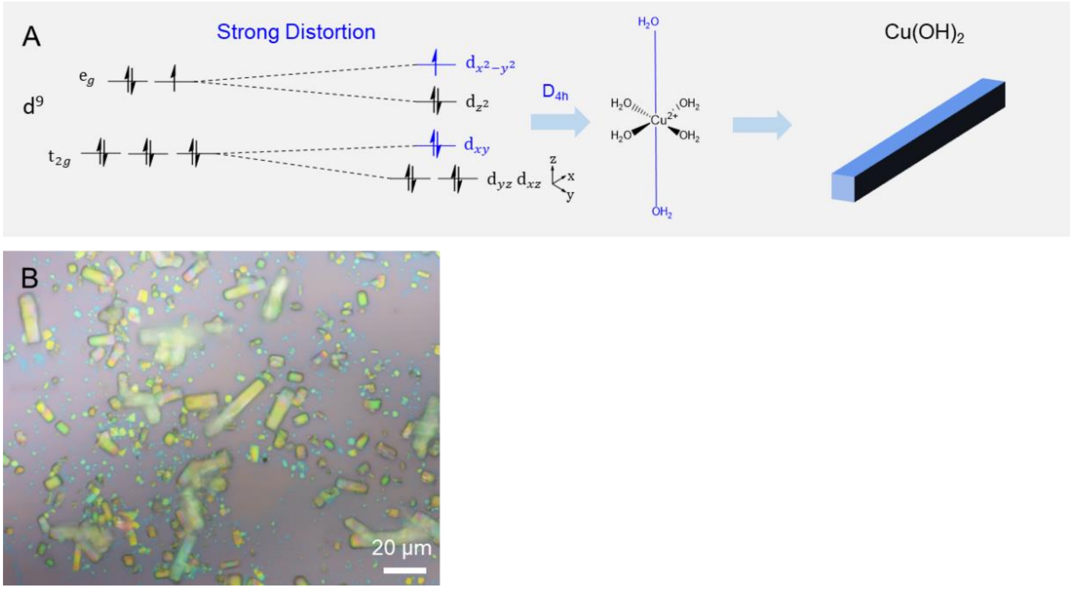

Fig. S6. Spin states directed geometry of $[Cu(H_2O)_6]^{2+}$ metal-ligand complex and the optical image of synthesized $Cu(OH)_2$ crystals . (**A**) $[Cu(H_2O)_6]^{2+}$ distorts to D_{4h} symmetry with dramatic elongation along the z-axis, yielding bulk crystals with stick-like morphology. (**B**) Optical image of synthesized $Cu(OH)_2$ crystals on SiO_2/Si substrate. No 2D flakes are observed.

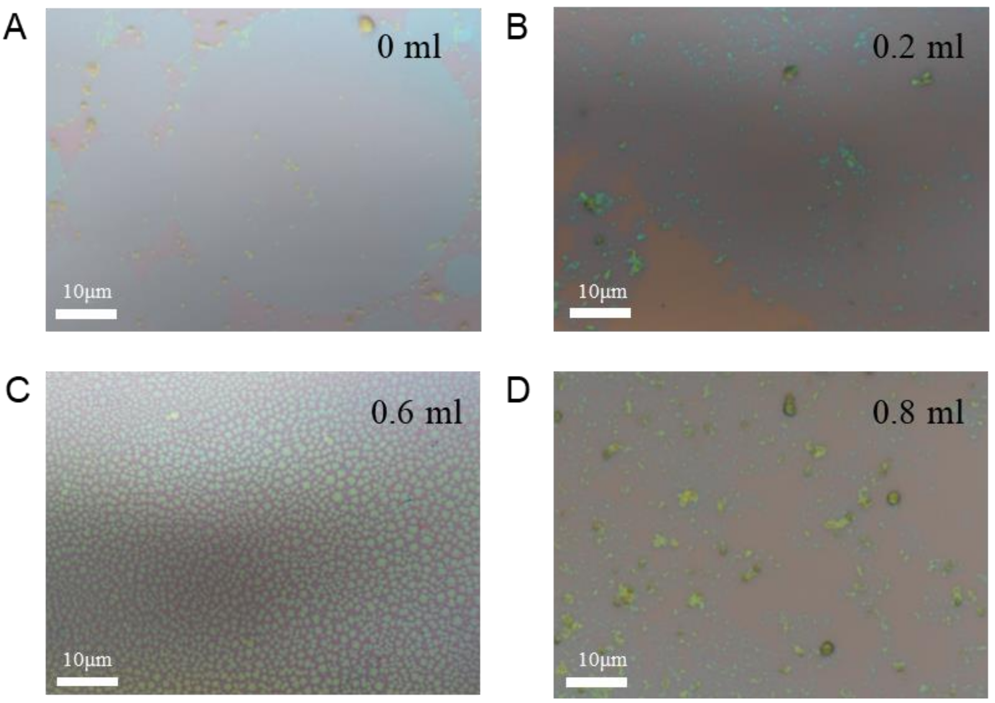

Fig. S7. $Ni(OH)_2$ morphology dependence on initial NH_4OH-tuned pH of solution from (**A**) no additional NH_4OH, (**B**) 0.2, (**C**) 0.6, and (**D**) 0.8 ml additional NH_4OH. The domain size decreases with the increasing NH_4OH, which will cease the 2D crystal expansion based on our proposed mechanism.

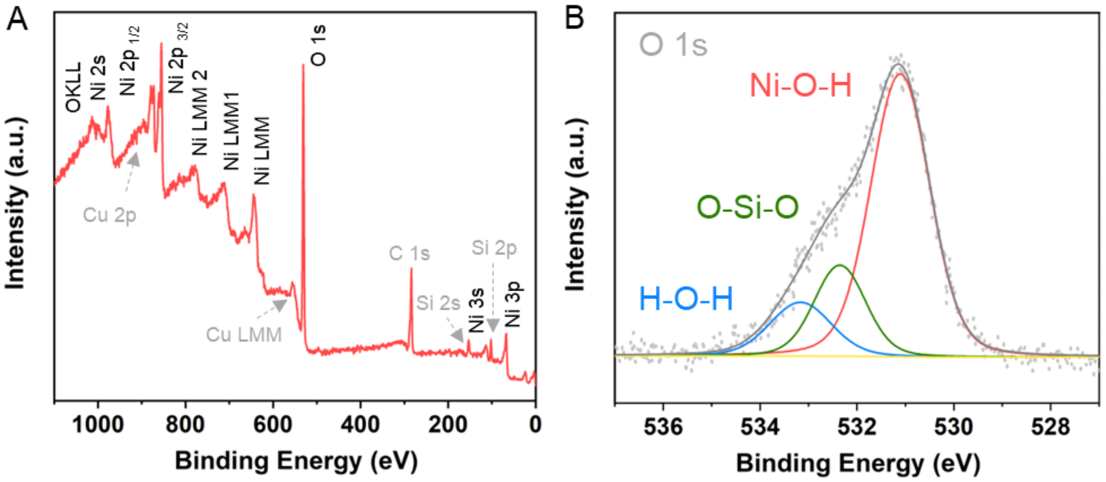

Fig. S8. Chemical states of synthesized 2D $Ni(OH)_2$, detected by XPS. (**A**) Survey spectrum, (**B**) O 1s

spectrum of $Ni(OH)_2$ thin flakes, indicating the occurrence of Ni and O elements at their binding energies and the appearance of H_2O, mainly as intercalation between layers to form α-$Ni(OH)_2$. Besides Ni and O signals from $Ni(OH)_2$ labeled in solid black, other extrinsic elements like C, Si and Cu are also found and labeled in dark grey in (**A**). The presence of C is normal in XPS examination since it is always part of the environment and it hardly can be excluded even under ultra-high vacuum. Moreover, Si is from the sample substrate and Cu is

from the sample stage of XPS. And the intensity of all these extrinsic elements is much lower than that of Ni and O.

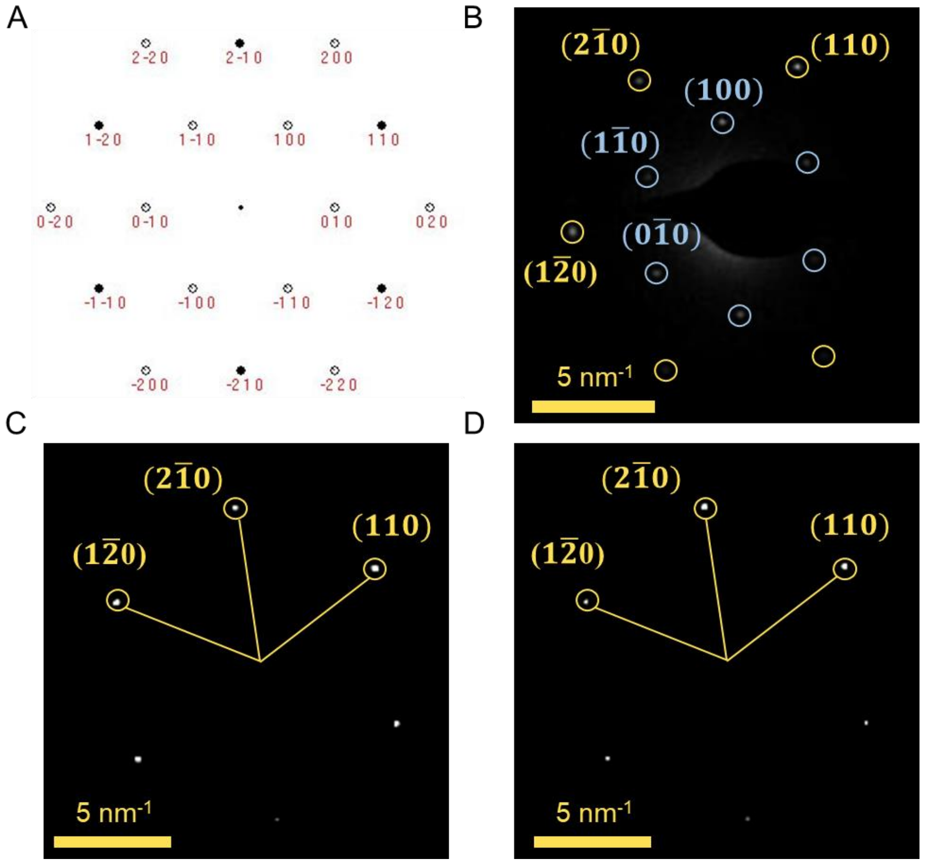

Fig. S9. SAED pattern of 2D Ni(OH)$_2$, detected by TEM. (**A**) Simulated diffraction of lattice planes within (220) from Stem-Cell. (**B**) Collected diffraction of lattice planes within (220). (**C**) Collected diffraction at a different position on the same flake. (**D**) Collected diffraction at a different position on the same flake, also is shown in Fig. 3C as inset.

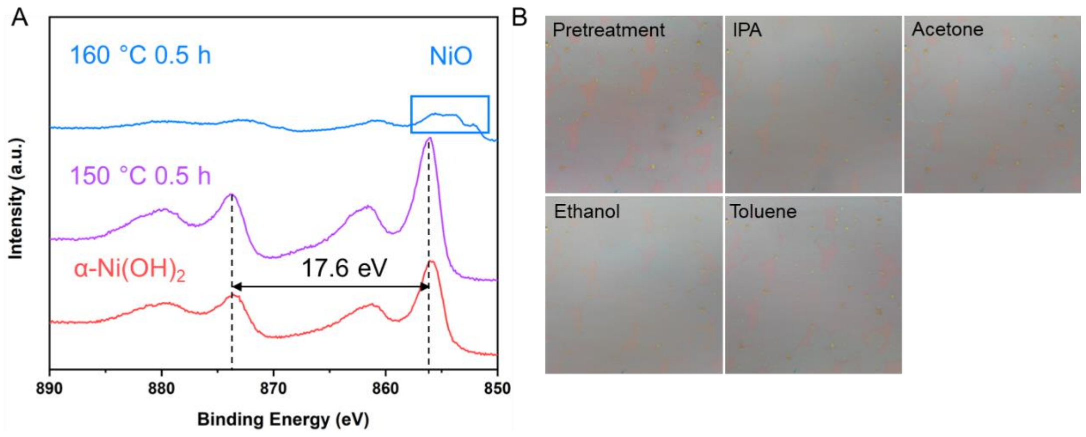

Fig. S10. Stability test of 2D α-Ni(OH)$_2$. (**A**) XPS spectra on 2D α-Ni(OH)$_2$ annealed under different temperatures for 0.5 hour, under 200 mTorr vacuum with 100 standard cubic centimeters per minute (sccm) Ar, suggesting good thermal stability of 2D α-Ni(OH)$_2$ under

150 °C. (**B**) Selected area of $Ni(OH)_2$ flakes on SiO_2/Si substrate before treatment and after 30 minutes immersion in IPA, acetone, ethanol and toluene, suggesting good stability in organic solvents.

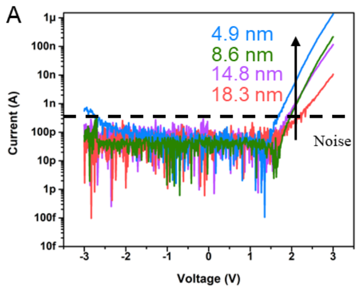

B

$Ni(OH)_2$ thickness (nm)	Breakdown Field (MV/cm)
4.9	4.47
8.6	2.92
14.8	1.73
18.3	1.64

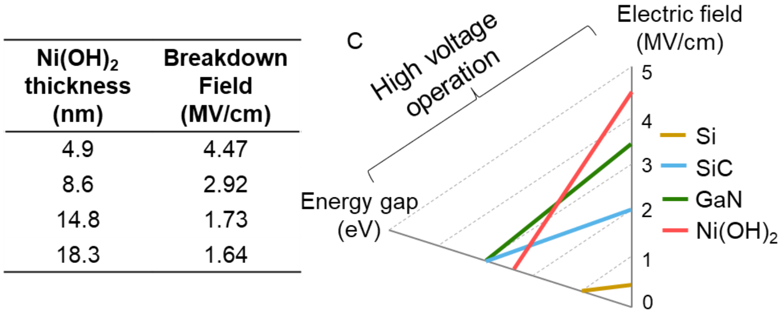

Fig. S11. Electrical Characterization of 2D α-$Ni(OH)_2$. (**A**) Current-voltage correlation. (**B**) List of the breakdown field of each flake. (**C**) Comparison of high voltage operation potential of $Ni(OH)_2$, Si, SiC, GaN, in terms of energy gap and electric field.

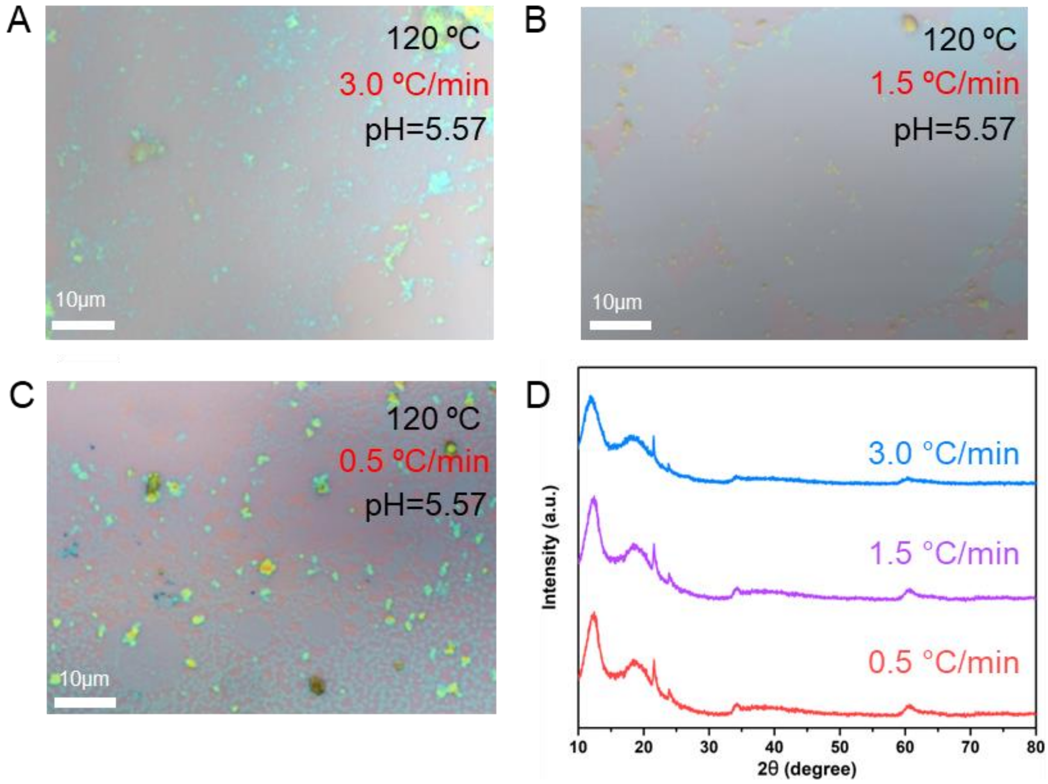

Fig. S12. $Ni(OH)_2$ morphology dependence on cooling rate from (**A**) 3.0 °C/min, to (**B**) 1.5 °C/min (the optimal condition) and (**C**) 0.5 °C/min, while the soaking temperature is 120 °C. (**D**) PXRD spectrum of $Ni(OH)_2$ synthesized under different cooling rate, suggesting cooling rate does not alter crystal phase.

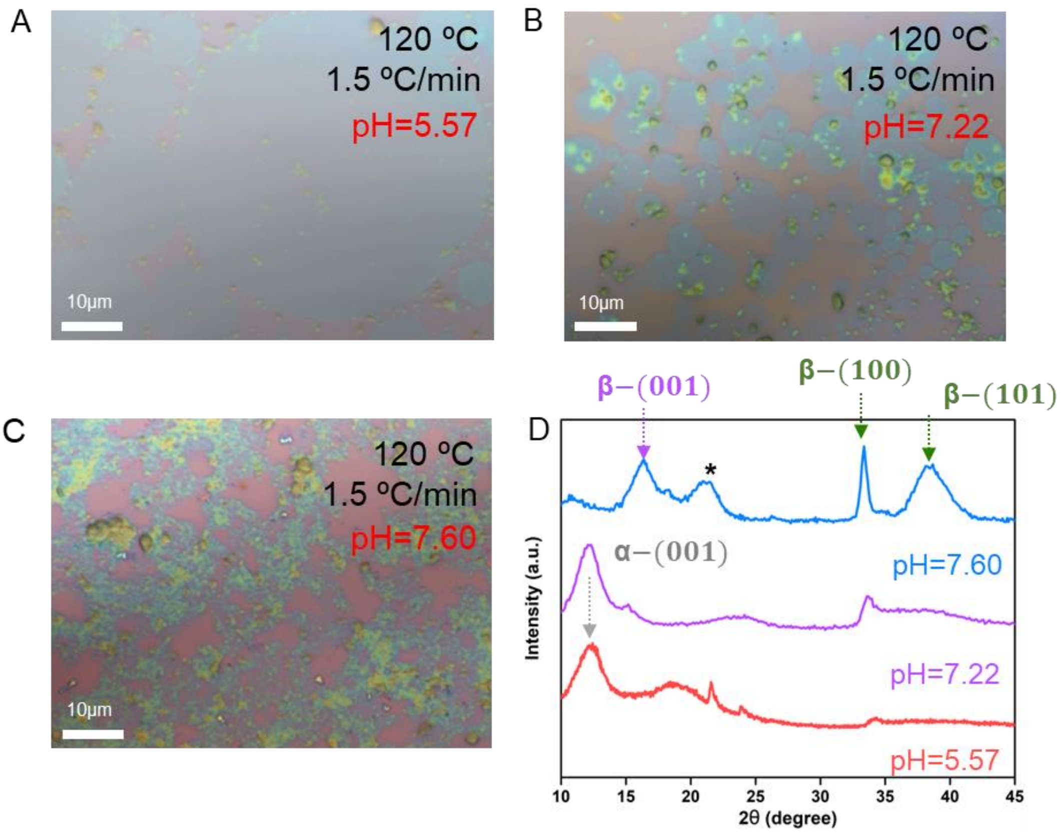

Fig. S13. $Ni(OH)_2$ morphology dependence on initial KOH-tuned pH of solution from (**A**) 5.57 (optimal condition, no KOH added) to (**B**) 7.60, (**C**) 7.22, while the soaking temperature is 120 ^oC and the cooling rate is 1.5 °C/min. (D) PXRD spectrum of $Ni(OH)_2$ synthesized with different starting pH, suggesting more β phase is formed with extra KOH addition. Peak labeled by "*" is generated from the stacking faults.

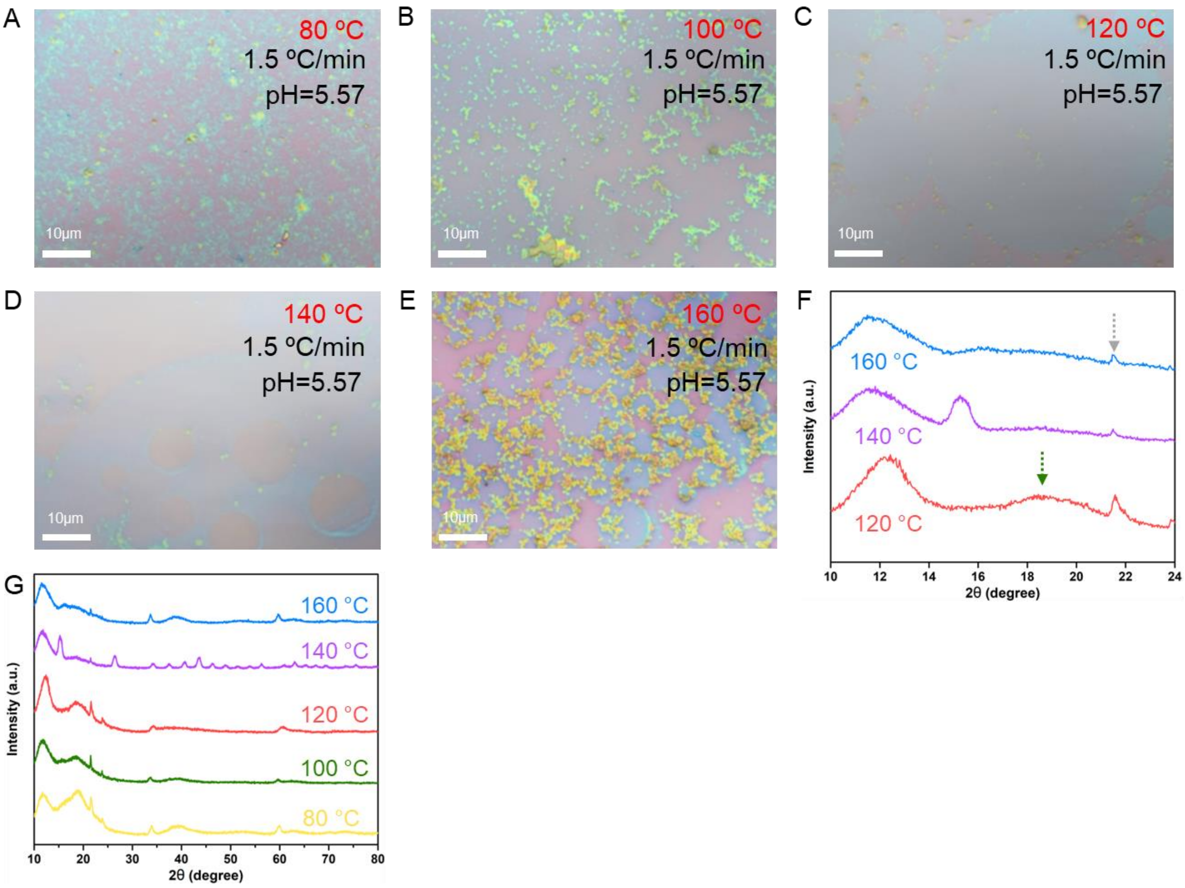

Fig. S14. $Ni(OH)_2$ morphology dependence on soaking temperature from (**A**) 80 °C to (**B**) 100 °C, (**C**) 120 °C (the optimal condition), (**D**) 140 °C and (**E**) 160 °C, while the cooling rate is 1.5 °C/min. (**F**) Phase variation with different soaking temperature, suggesting higher purity of α phase is synthesized under higher temperature. (**G**) Whole PXRD spectra along 2θ=10-80 ° of $Ni(OH)_2$ synthesized from different soaking temperature.

REFERENCES

1. Huang, L. F., Hutchison, M. J., Santucci, R. J., Scully, J. R. & Rondinelli, J. M. Improved Electrochemical Phase Diagrams from Theory and Experiment: The Ni-Water System and Its Complex Compounds. *J. Phys. Chem. C* **121**, 9782–9789 (2017).

2. Hall, D. S., Lockwood, D. J., Bock, C. & MacDougall, B. R. Nickel hydroxides and related materials: A review of their structures, synthesis and properties. *Proc. R. Soc. A Math. Phys. Eng. Sci.* **471**, (2015).

3. Park, H. W., Chae, J. S., Park, S. M., Kim, K. B. & Roh, K. C. Nickel-based layered double hydroxide from guest vanadium oxide anions. *Met. Mater. Int.* **19**, 887–894 (2013).

4. Zhao, Y. *et al.* Preparation of Ni(OH)2 nanosheets on Ni foam via a direct precipitation method for a highly sensitive non-enzymatic glucose sensor. *RSC Adv.* **5**, 53665–53670 (2015).
